# The Elusive Biological Activity of Scorpionates: A Useful Scaffold for Cancer Therapy?

**DOI:** 10.3390/molecules29235672

**Published:** 2024-11-30

**Authors:** Artem Petrosian, Pedro F. Pinheiro, Ana P. C. Ribeiro, Luísa M. D. R. S. Martins, Gonçalo C. Justino

**Affiliations:** 1 Centro de Química Estrutural—Institute of Molecular Sciences, Instituto Superior Técnico, Universidade de Lisboa, Av. Rovisco Pais, 1, 1049-001 Lisboa, Portugal; artem.petrosian@tecnico.ulisboa.pt (A.P.); pedro.pinheiro@tecnico.ulisboa.pt (P.F.P.); apribeiro@tecnico.ulisboa.pt (A.P.C.R.); 2 Escola Superior de Tecnologia do Barreiro, Instituto Politécnico de Setúbal, Rua Américo da Silva Marinho, 2839-001 Lavradio, Portugal; 3 Departamento de Engenharia Química, Instituto Superior Técnico, Universidade de Lisboa, Av. Rovisco Pais, 1, 1049-001 Lisboa, Portugal

**Keywords:** scorpionate complexes, metal-based drugs, cytotoxicity, anticancer lead compounds

## Abstract

Cancer remains a formidable challenge, requiring the constant pursuit of novel therapeutic agents and strategies. Scorpionates, known for their unique coordination properties, have recently gained attention for their anticancer potential. Traditionally applied in catalysis, these compounds have demonstrated notable cytotoxicity across various cancer cell lines, often surpassing the efficacy of conventional chemotherapeutics. This review addresses recent findings on scorpionate complexes, emphasizing the impact of metal choice and ligand design on biological activity. Copper and ruthenium scorpionates show promise, leveraging redox activity and mitochondrial disruption mechanisms to selectively induce cancer cell death. Ligand modifications, including sulfur-containing heterocycles and unsubstituted pyrazoles, have proven effective in enhancing cytotoxicity and selectivity. Furthermore, dipodal ligands show unique potential, with selective binding sites that improve stability and facilitate specific cellular interactions, such as targeting metastatic pathways. These findings highlight the largely unexplored potential of scorpionate complexes, positioning them as candidates for next-generation anticancer therapies. Continued research into structure–activity relationships and precise mechanisms of action could pave the way for developing highly potent and selective anticancer agents based on scorpionate chemistry.

## 1. Introduction

Cancer is a complex, multifactorial disease characterized by intricate and profound cellular transformations at the molecular level, often triggered by genetic mutations and other biochemical alterations. These molecular changes lead to a state of uncontrolled cellular growth, proliferation, and division, a defining feature of neoplastic growth, which can cause the rapid accumulation of tissue mass [1,2]. Under typical conditions, senescent or damaged cells receive intercellular signals to undergo programmed cell death (apoptosis), allowing for replacement with healthy cells. However, cancer cells are able to evade apoptosis through different mechanisms such as the upregulation of anti-apoptotic proteins like the Bcl-2 family members, immune escape, and deficiencies in mitochondrial-mediated apoptosis pathways [3]. As a result, cancer cells exhibit a much longer lifespan and uncontrolled proliferation, diverting essential nutrients and resources from non-tumoral cells.

The development of effective cancer therapeutics remains one of the most significant challenges in modern medicine, with researchers continuously seeking novel approaches to target cancer cells while minimizing damage to healthy tissues. Traditional chemotherapeutic agents, while effective in many cases, often lead to severe side effects due to their inability to discriminate between rapidly dividing normal cells and cancer cells [4]. This has driven the search for more selective therapeutic approaches that exploit the unique characteristics of cancer cells, including their altered metabolism, specific surface markers, and dysregulated signaling pathways [4,5].

Recent advances in molecular biology and drug discovery have led to the emergence of targeted therapies that show promising results in various cancer types. These include small-molecule inhibitors, monoclonal antibodies, and innovative drug delivery systems that can specifically target cancer cells [6].

**Figure 1:**
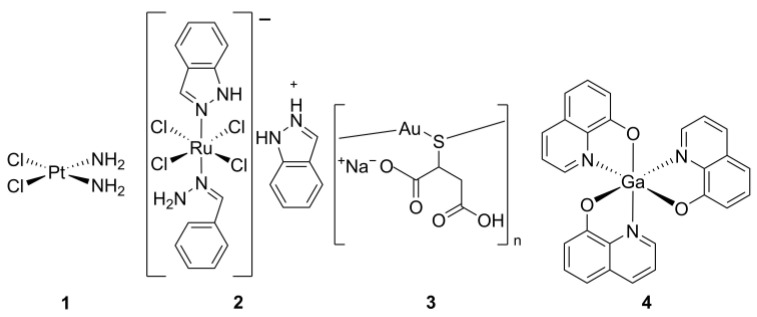
Structures of coordination complexes used in cancer therapy.

Coordination complexes such as cisplatin (**1**, *cis*-diamminodichloroplatinum(II)), KP1019 (**2**, indazole *trans*-[tetrachlorobisruthenate(III)]), aurothiomalate (**3**), KP46 (**4**, tris-(8-quinolinolato)gallium(III)), and many others have already been successfully used to treat different forms of cancer [7] (Figure 1). The successful history of cisplatin, discovered in 1966 to be a potent trans-domain cell division inhibitor and used either alone or as a co-adjuvant almost as a go-to anticancer drug, has recently led to an intensive exploration of novel prototypical cancer drugs [8,9]. While these, and other, metal complexes have been included in clinical trials as prospective anticancer agents in solid tumors [10,11,12], none have been approved for cancer treatment, as is the case of cisplatin.

Within the domain of metal complexes, some coordination compounds with the tri-dentate scorpionate ligand were found to exhibit interesting anticancer activity [13,14]. The chemistry of these compounds has been studied since 1966, allowing for the generation of a considerable number of scorpionate-shaped ligands and their complexation to different metal atoms [15]. The straightforward synthesis of diverse compounds within the scorpionate family, combined with preliminary findings indicating notable anticancer potential, positions the scorpionate scaffold as a compelling focus in medicinal chemistry.

In the last few years, substantial progress has been made in investigating the anticancer potential of various scorpionate complexes, revealing their significant cytotoxic effects across different cancer cell lines. Herein, we discuss and underscore the promise of scorpionate–metal complexes as versatile, selective, and effective alternatives to conventional chemotherapeutic agents, suggesting their potential to form the basis of next-generation anticancer therapies.

The anticancer potential of metal complexes has been recognized since the discovery of cisplatin, a milestone that inspired extensive research into metal-based therapeutics [12].

Essential metals such as zinc, copper, and iron play pivotal roles in numerous biological processes within the human body [16]. A key feature of their antiproliferative properties is the capacity to generate reactive oxygen species (ROS) via Fenton chemistry. Cancer cells, characterized by accelerated metabolism, accumulate ROS more rapidly than normal cells, leading to cellular stress and, ultimately, cell death as homeostasis is disrupted [16,17,18]. This ROS-mediated cytotoxicity is a central mechanism in the activity of metal complexes against cancer. Similarly, scorpionate complexes have also demonstrated anticancer properties, offering promising coordination chemistry platforms for therapeutic exploration [19,20,21].

## 2. Scorpionate Chemistry

**Figure 2:**
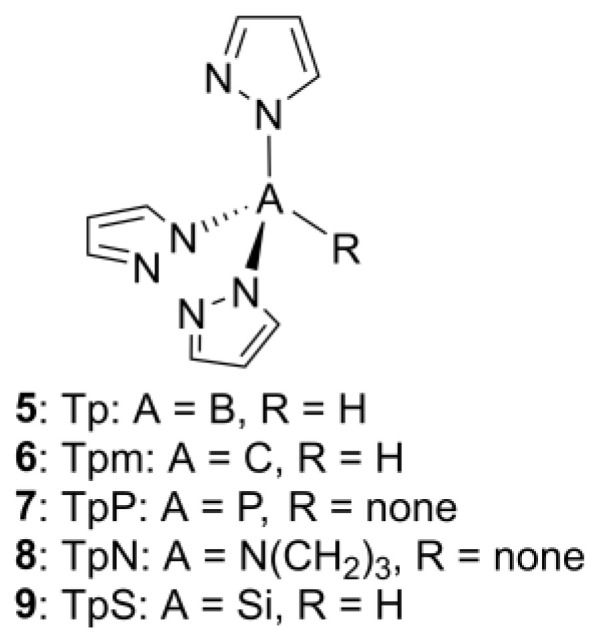
The structure of the prototypical Tp ligand and derivative members of the homo- and heteroscorpionate families.

Scorpionate ligands have been known for more than five decades since Trofimenko synthesized and reported this new type of tridentate ligand in 1967 [22]. The peculiar name of the ligands is due to the similarity between their metal-coordinating geometry and the way in which scorpions attack their prey with pincers and sting. The first synthesized family of scorpionates was the anionic hydrotris(pyrazol-1-yl)borates (Tp, **5**, Figure 2). Afterwards, different scorpionates with substituted pyrazole rings and boron atoms were synthesized, leading to different steric and electronic effects [23].

Moreover, the pyrazolyl rings can be replaced with any other azolyl heterocycles that may contain nitrogen, oxygen, or sulfur atoms [24]. The replacement of one of the heterocycles with any other coordinating or non-coordinating group is possible as well, leading to the so-called heteroscorpionates that comprise a combination of different azolyl rings or their replacement by other coordinating or non-coordinating functions (e.g., hydride, alkyl or aryl, acetate, etc.), as opposed to homoscorpionates, where donor atoms come from equivalent moieties.

The central atom of scorpionate ligands can also be changed, resulting in several congeners (Figure 2): tris(pyrazol-1-yl)methanes (Tpm, **6**), tris(pyrazol-1-yl)phosphanes (TpP, **7**), tris(pyrazol-1-yl)amines (**8**), and tris(pyrazol-1-yl)silanes (TpS, **9**) [24,25,26,27]. This large variety of different substituents, heterocycles, and central atoms that can be used makes scorpionates a very diverse family of ligands with different properties and activities.

All of these different scorpionate ligands were reported to form different coordination compounds from alkaline to transition metals [28,29,30,31,32,33,34,35,36]. Moreover, tris(pyrazol-1-yl)borates were shown to complex with lanthanides and actinides [36,37,38]. These coordination compounds have a widespread use, starting from their applications as catalysts for polymerization, oxidation, and nitrene transfer reactions to bioorganic and medicinal chemistry for modeling enzyme, antimicrobial, antioxidant, and anticancer activity [19,23,35,39,40,41,42]. In comparison to the great advances made in the use of scorpionates as catalysts, their activity, as anticancer agents, is still rather unexplored and far from systematized. This fact, along with the wide diversity of scorpionates, their properties, and the severe need for new potent compounds with anticancer activity, makes the exploration of scorpionates’ potential biological activity against cancer a very promising and interesting research theme.

## 3. Poly(pyrazol-1-yl)borate Complexes

**Figure 3:**
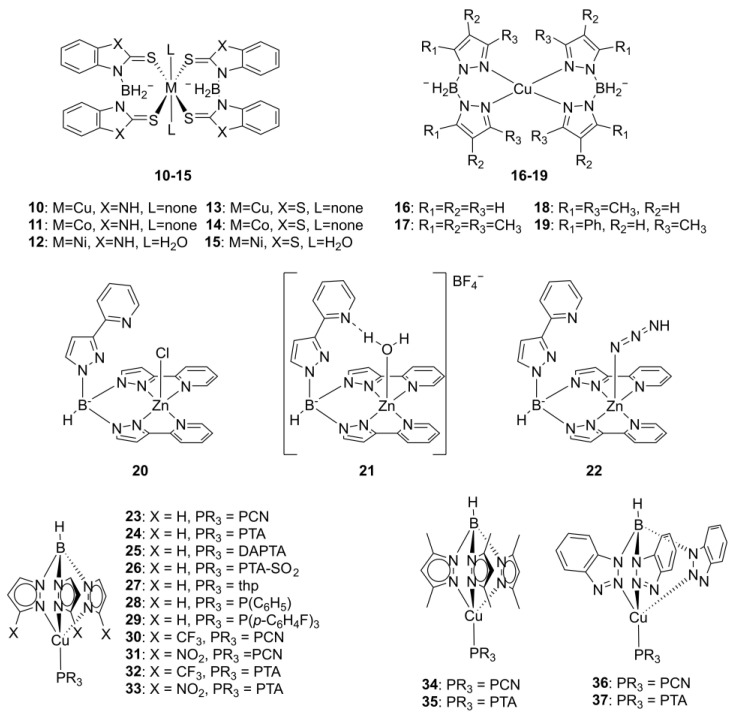
Structure of scorpionate complexes with poly(pyrazol-1-yl)borate ligands. Adapted from [21,43,46,47].

Full-sandwich bis-scorpionate complexes with Cu(II), Ni(II), and Co(II) ions and 2-mercaptobenzimidazole and 2-mercaptobenzothiazole heterocycles, replacing the conventional pyrazole ligands (**10**–**15**, Figure 3) [43], have been produced as these heterocycles have established biological activity [44,45] and also due to sulfur’s role as a soft donor atom, which enhances coordination stability with transition metals such as copper, potentially augmenting the complexes’ robustness and bioactivity.

**Figure 4:**
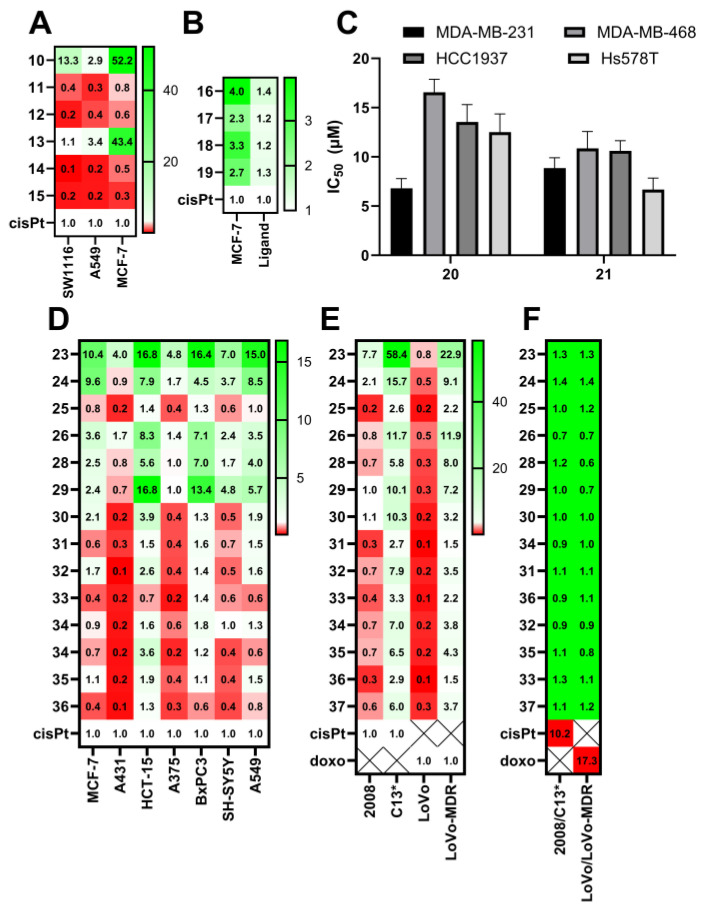
The cytotoxicity of the poly(pyrazol-1-yl)borate complexes discussed in the text determined by the generic MTT assay. (**A**) The relative cytotoxicity of compounds **10**–**15**, computed from the values of Faghig et al. [43]. (**B**) The relative cytotoxicity of compounds **16**–**19**, computed from the values of Ghorbanpour et al. [46]. (**C**) The cytotoxicity of **20** and **21**, from Narwane et al. [21]. (**D**) The relative cytotoxicity in tumor cell lines of compounds **23**–**37**, computed from the values of Gandin et al. [47]. (**E**) The relative cytotoxicity in paired cell lines of **23**–**37**; the ovarian cancer 2008 and C13* cell lines are cisplatin-sensitive and cisplatin-resistant, respectively, while the LoVo and LoVo-MDR cell lines are colon cancer cell lines, with the latter expressing a multidrug-resistance phenotype. This is computed from the values of Gandin et al. [47]. (**F**) The resistance factor of 23–37, computed from Gandin et al. [47]. The relative cytotoxicity was computed from the results published in the original works as the ratio of IC_50_ for cisplatin divided by IC_50_ for the complex; the higher the value, the higher the activity of the complex (relative to cisplatin, white). Values in red are less active than cisplatin, while values in green are more active than cisplatin. cisPt—cisplatin, doxo—doxorubicin; MDR—multidrug-resistant. The original cytotoxicity data are given in the Supplementary Materials (Tables S1–S5).

The anticancer activity of complexes **10**–**15** was assessed against colon (SW116), lung (A 549), and breast carcinoma (MCF-7) cancer cell lines, with the results compared to the established chemotherapy drug, cisplatin. Notably, the complexes exhibited the highest activity against colon cancer cells. Among these, only the copper-based scorpionates demonstrated a cytotoxicity superior to that of cisplatin (Figure 4A; original cytotoxicity data given in Supplementary Materials, Table S1). Specifically, **10** exhibited the most potent activity, with IC_50_ values of 0.65 μM, 11.44 μM, and 1.18 μM against the SW116, A549, and MCF-7 cell lines, respectively, while cisplatin exhibited IC_50_ values of 8.62 μM, 33.52 μM, and 61.56 μM for the same cell lines. Moreover, complexes containing 2-mercapto-benzimidazole displayed significantly higher anticancer activity compared to those with 2-mercapto-benzothiazole. A further analysis of the mechanism of cell death revealed that the antiproliferative/cytotoxic effect associated with compound **10** is partly mediated through apoptosis induction in cancer cells [43].

To further evaluate the scorpionates’ activity, DNA interaction and genotoxicity assays using comet and mobility shift assays along with molecular docking studies were carried out. Both the comet and mobility shift assays demonstrated that the copper scorpionate **10** interacted with DNA in a manner similar to cisplatin, indicating potential DNA intercalation capabilities, predominantly at the minor groove of the DNA, involving the formation of hydrogen bonds, as suggested by molecular docking. Although, like cisplatin, **10** intercalates DNA, it does not recapitulate the DNA base coordination of the platinum ion, which is observed with cisplatin.

Although complexes **10**–**15** (as well as **16**–**19**, Figure 3) do not feature scorpionate ligands but rather chelating ligands, their pyrazolyl-, imidazolyl-, and thiazolyl-bearing BX_2_H_2_^–^ ligands, with their demonstrated biological activity, position them as an entry point for exploring the anti-tumoral activity of scorpionate coordination complexes.

Recently, another investigation into the anticancer properties of copper-based full-sandwich bis(pyrazolyl)borate complexes was conducted [46]. In this study, four different copper(II) complexes (**16**–**19**) were synthesized using pyrazolyl, 3,5-dimethylpyrazol-1-yl, 3,4,5-trimethylpyrazol-1-yl, and 3-methyl-5-phenylpyrazol-1yl ligands.

The cytotoxic effects of these complexes were evaluated against the breast carcinoma (MCF-7) cell line and compared to cisplatin. Complexes bearing more substituted and bulkier ligands exhibited reduced activity compared to the unsubstituted ones; the unsubstituted full-sandwich copper(II) bis(pyrazolyl)borate complex **16** exhibited the highest activity, with an IC_50_ value of 25.37 μM, whereas the least active was the copper(II) bis(3,4,5-trimethylpyrazolyl)borate complex **18**, with an IC_50_ value almost twice as high at 44.21 μM. Nevertheless, all of the complexes demonstrated higher activity than cisplatin, with complex **16** exhibiting an activity nearly four times higher than that of cisplatin (Figure 4B) [46]. A structure–activity approach revealed that the dipole moment displayed the most significant direct correlation with the IC_50_ value, as a decrease in the dipole moment increases the lipophilicity of these compounds, influencing their biological properties, partition, and anticancer activity. Molecular docking simulations also suggest that complex **17** interacts with cyclin-dependent kinase 2 (CDK2, binding energy of −7.21 kcal/mol) and epidermal growth factor receptor (EGFR, binding energy of −6.62 kcal/mol), with −7.65 and −6.62 kcal/mol for the controls (the co-crystalized inhibitors) [46].

Beyond copper, zinc–scorpionate complexes (**20**–**22,** Figure 3) bearing tris-(2-pyridyl)-(pyrazol-1-yl)borate ligands have also been found to display IC_50_ values roughly half of those of cisplatin against triple-negative breast cancer lines MDA-MB-231, MDA-MB-468, HCC1937, and Hs578T [21]. Triple-negative breast cancer (TNBC) is characterized by the absence of three key receptors in cancer cells—estrogen receptor (ER), progesterone receptor (PR), and human epidermal growth factor receptor 2 (HER2)—making TNBC more difficult to treat. Also, TNBC is more aggressive than other types of breast cancer, tending to proliferate quickly and being more likely to metastasize. These challenges underscore the need for novel therapeutic strategies.

Although zinc does not exhibit redox activity like the previous transition metals, it is still an essential element in various cellular pathways, including signaling, maintaining homeostasis, and modulating cytotoxicity [48,49]. These factors can ultimately influence the overall activity of these complexes.

The binding efficacy of zinc complexes with calf thymus DNA was evaluated, showing that one complex molecule binds three to four base pairs, with complex **20** demonstrating the lowest binding energy, likely due to the replacement of a chlorine group by a hydroxyl group upon cellular entry. Molecular docking studies revealed that complex **20** exhibited the lowest binding free energy, at −10.3 kcal/mol, indicating strong intercalation with DNA. Additionally, interactions with bovine serum albumin (BSA) were analyzed, revealing increased absorption intensity and significant decreases in fluorescence intensity, suggesting static quenching and confirming the complexes’ interactions with BSA.

Despite their redox inactivity, zinc–scorpionate complexes, being able to bind DNA and proteins, could represent a promising class of therapeutic agents for targeting cancer, particularly in challenging contexts such as triple-negative breast cancer. Their ability to interact with biological macromolecules may enhance their cytotoxic effects, providing a potential alternative to traditional chemotherapeutics. However, it must be noted that the complexes mentioned in this section have not been tested against control cell lines, but only using cancer cell lines as candidate drug leads with increased activity relative to a standard, usually cisplatin (Figure 4C).

Building on the synthetic versatility of the pyrazole ring, a series of Cu(I) scorpionate complexes with tris(pyrazolyl)ligands was prepared, introducing pyrazolyl modifications (**23**–**37**, Figure 3) [47], namely the introduction of CF_3_ and NO_2_ groups at position 3 of the pyrazolyl group. Other derivatives also feature the replacement of this ring by a benzo-1,2,3-traizole moiety. These complexes, with various Cu-coordinating ligands beyond the tp ligands, were assessed for their anti-tumoral activity in a panel of breast cancer (MCF-7), cervical cancer (A431), colon cancer (HCT-15), pancreatic cancer (BxPC3), lung (A549) cancer, neuroblastoma (SH-SY5Y), and melanoma (A375) cell lines. The introduction of either electron-donating (CH_3_) or electron-withdrawing (NO_2_ and CF_3_) groups in the tris(pyrazolyl)borate ligand significantly weakened the cytotoxicity of the Cu complex. A 9- to 11-fold reduction in the in vitro anti-tumor activity was observed upon the introduction of these groups (**30**, **31**, and **34** vs. **23**, for the PCN ligand; and **32**, **33**, and **35** vs. **24**, for the PTA ligand). The introduction of the benzotriazolyl group also led to a 7- to 20-fold activity decrease (**36** and **37** vs. **23** and **24**, respectively) [47]. Complexes **23** and **24**, bearing non-modified pyrazolyl rings, were the most active (Figure 4D).

These Cu(I) complexes were also tested for their in vitro anti-tumor activity using two human cancer cell line pairs: ovarian cancer cells (2008/C13*), selected for cisplatin sensitivity/resistance, and colon cancer cells (LoVo/LoVo MDR), which exhibit a multidrug-resistant (MDR) phenotype. The cytotoxicity profiles were similar for these cell lines (Figure 4E). Interestingly, for the 2008/C13* cells, the resistance factor (RF), computed as the ratio of the IC_50_ values for the resistant and the sensitive cell lines, were 6–10 times lower than for cisplatin, indicating no cross-resistance (Figure 4F). Similarly, for LoVo/LoVo MDR cells, RFs were on average 30 times lower than those for doxorubicin, suggesting that these compounds are not MDR substrates [47].

The cytotoxicity of the two most active complexes of this set (**23** and **24**) were also tested against rapidly proliferating non-tumoral human embryonic kidney HEK293 cells and displayed IC_50_ values about four times higher than cisplatin, but also displayed a selectivity index about three times higher than that of cisplatin, suggesting a preferential cytotoxicity of **23** and **24** toward neoplastic cells [47].

## 4. Poly(pyrazol-1-yl)methane Complexes

Tris(pyrazolyl)methane (tpm) ligands are neutral analogues of the commonly used anionic tris(pyrazolyl)borates and are formally derived by replacing the apical [BH]− with a C-R group. As they are boron analogues, tpm ligands act as six-electron donors and have been show to form coordination complexes with metals from groups 1 to 14 [50,51]. Tpm ligands often feature substituted pyrazole rings, most frequently 3-, 4-, or 5-methyl-, 3,5-dimethyl-, and 3,4,5-trymethylpyrazole, but substitution with phenyl and *t*-Bu groups is also frequent 51], and many of the complexes discussed here feature these substitutions.

**Figure 5:**
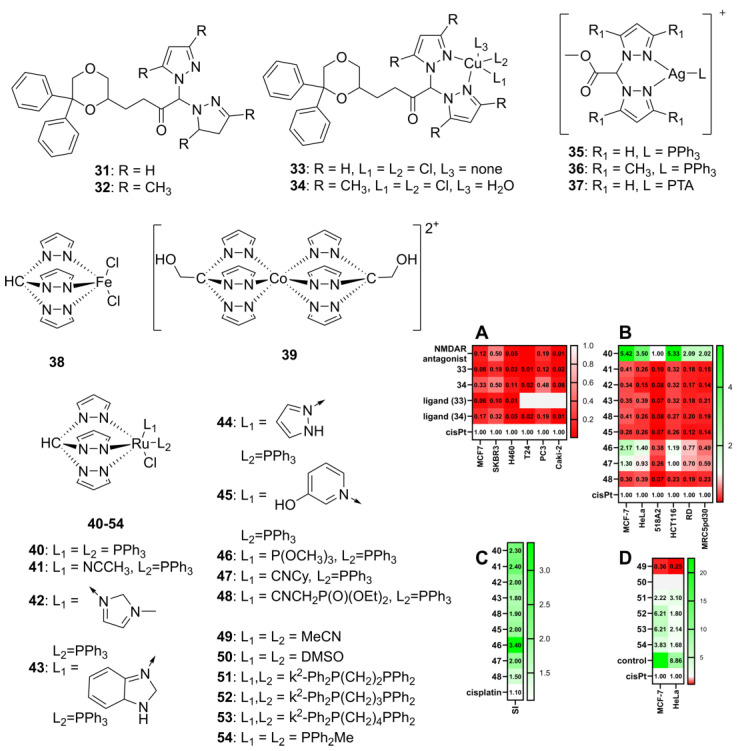
The structures of scorpionate complexes with poly(pyrazol-1-yl)methane ligands, with cytotoxicity values determined by the MTT assay. Adapted from [52,53,54,55]. (**A**) The relative cytotoxicity of compounds **33** and **34**, computed from the values of Morelli et al. [52]. (**B**) The relative cytotoxicity of compounds **40**–**48**, computed from the values of Cervinka et al. [55]. (**C**) The selectivity index of compounds **40**–**48**, from Cervinka et al. [55], computed as the ratio of IC50 for the non-tumoral MRC5pd30 cell line divided by the average of IC50 for the remaining cell lines. (**D**) The relative cytotoxicity of compounds **49**–**54**, computed from the values of Walker et al. [56]. The relative cytotoxicity was computed from the results published in the original works as the ratio of IC50 for cisplatin divided by IC50 for the complex; the higher the value, the higher the activity of the complex (relative to cisplatin, white). Values in red are less active than cisplatin, while values in green are more active than cisplatin. cisPt—cisplatin. The original cytotoxicity data are given in the Supplementary Materials (Tables S6–S8).

The ability of scorpionate-like copper(II) complexes with bis(pyrazol-1-yl)methane ligands functionalized with a known antagonist of the *N*-methyl-D-aspartate (NMDA) receptor (**31**–**34**, Figure 5), effectively replacing the third pyrazole with a bioactive molecule, was investigated by Morelli et al. [52].

Certain breast cancer cell lines express the NMDA and NMDAR2 receptors, which play a critical role in breast cancer progression [55,57]. Aligned with this, the authors aimed to explore the potential therapeutic efficacy of combining the mechanisms of a noncompetitive NMDA antagonist with those of a copper scorpionate against a panel of human prostate (PC3), breast cancer (MCF7 and SKBR3), non-small-cell lung cancer (H460), bladder cancer (T24), and renal cancer (Caki2) cell lines.

While the precursors bis(pyrazol-1-yl)acetate and bis(3,5-dimethyl-pyrazol-1-yl)acetate exhibited no significant activity on their own, their conjugates with an NMDA antagonist (**31**–**32**) demonstrated enhanced activity within the micromolar range. The study revealed differences in efficacy among the conjugated derivatives. The bis-pyrazol-1-yl conjugate **31** showed lower cytotoxicity than the standalone NMDA antagonist, while the second derivative, **32**, not only matched but, in some assays, surpassed the antagonist’s activity—particularly against T24 and Caki2 cell lines. Among the copper scorpionates, complex **33**, which contains unsubstituted pyrazolyl rings, demonstrated moderate efficacy across various cell types. In contrast, complex **34** exhibited a significantly higher cytotoxic effect, outperforming both previous ligands and other complexes. These findings suggest that unsubstituted pyrazolyl rings may reduce anticancer activity in these complexes.

Furthermore, the mechanism of action of complex **34** against the MCF7 cell line was found to involve an increase in ROS production and the induction of oxidative stress, which translated into an increase in the mitochondrial membrane potential. A Western blot analysis revealed upregulated expression of the immunoglobulin heavy chain binding protein, indicating the induction of endoplasmic reticulum (ER) stress. These cellular changes are characteristic of paraptosis [58], suggesting that complex **34** may induce paraptotic cell death pathways. This is particularly relevant as breast cancer cells often exhibit resistance to apoptosis, and the ability of complex **34** to induce paraptosis highlights its potential as a novel therapeutic agent.

Bis(pyrazol-1-yl)acetate complexes with silver(I) (**35**–**37**, Figure 5A) have also been investigated for their potential use as agents in anticancer treatment [53], using both 2D and 3D cell culture models of human colon cancer (HCT-15), pancreatic cancer (PSN-1), cervical cancer (A431), breast cancer (MDA-MB-231), ovarian cancer (2008), cisplatin-resistant ovarian adenocarcinoma (C13), and small-cell lung cancer (U1285) solid tumor cell lines.

All three silver complexes demonstrated significant cytotoxicity in 2D cultures in the low micromolar range, outperforming cisplatin. The U1285 and cisplatin-resistant C13 cell lines were particularly susceptible, with the silver complexes showing up to 14-fold greater potency than cisplatin in some cases. Notably, complex **36**, with a 3,5-dimethylpyrazolyl ring, exhibited superior activity compared to the unsubstituted pyrazolyl complex **27**, although even the latter outperformed cisplatin against HCT-15, MDA-MB-231, U1285, and C13 cell lines. In the U1285 3D culture model, the three complexes have IC_50_ values of 63.8, 27.9, and 22.0 µM, respectively (vs. 65 µM for cisplatin). This highlights the promising potential of silver(I) scorpionate complexes as anticancer agents, especially in drug-resistant cancer cells.

Cellular uptake studies also showed that **36** and **37**, the more active complexes, accumulated in higher quantities in the cells. As no significant difference in either activity of uptake levels was found between **36** and **37**, which feature, respectively, 1,3,5-triaza-7-phosphaadamantane (PTA) and the triphenylphosphine (PPh_3_) ligands, this suggests that the primary factor contributing to the uptake and efficacy of the complexes is the lipophilicity of the bidentate bis(pyrazol-1-yl)acetate ligand.

Cancer cells often exhibit high levels of thioredoxin reductase (TrxR) to manage oxidative stress in the tumor microenvironment [59]. Building on the widely described TrxR inhibitor activity of Ag(I) complexes, complexes **35**–**37** (Figure 5) were evaluated for their ability to inhibit TrxR activity both in cell-free systems and in U1285 cells. As TrxR plays an essential role in cellular redox homeostasis, the effect of complexes **35**–**37** on total cellular sulfhydryl content and on ROS production was also assayed.

While in the cell-free assay these complexes displayed an activity lower than that of the reference TrxR inhibitor auranofin, in intact U1285 cells, complexes **36** and **37** displayed an inhibitory activity similar to that of auranofin. The three complexes also led to increased cellular hydrogen peroxide.

In addition to inhibiting TrxR, silver complexes **35**–**37** were found to be able to modulate total thiol content. In particular, the reduction in cellular sulfhydryl content obtained with **37** at the higher concentration tested (3 µM) was very similar to that induced by equimolar auranofin. These complexes also caused a substantial time- and dose-dependent increase in cellular basal hydrogen peroxide production, a hallmark of oxidative stress. However, this was less pronounced than the hydrogen peroxide production elicited by antimycin, a classic inhibitor of the mitochondrial respiratory chain at the level of complex III.

The impact of ROS on mitochondria was evaluated through mitochondrial membrane potential analysis, revealing that treatment with the silver complexes caused mitochondrial hypopolarization, with up to 30% hypopolarization in cells treated with complex **36**. Consistent with previous trends, **36** and **37**, bearing the 3,5-dimethylpyrazole moiety, exhibited higher activity. Transmission electron microscopy further confirmed the anti-mitochondrial effects, showing significant mitochondrial swelling, reinforcing the evidence of the ability of these complexes to induce mitochondrial disruption.

Tris-substituted (pyrazol-1-yl)methanes and its congeners have also been studied for their biological activity [54]. Dichlorotris(pyrazol-1-yl)methane iron(II) (**38**) and bis(2,2,2-tris(pyrazol-1-yl)ethanol)cobalt(II) (**39**) (Figure 5) were analyzed for their cytotoxicity, motogenicity, and effect on the metabolome on model B16 (mouse epithelial skin melanoma) and HCT116 cancer cell lines, as well as on the non-tumoral cell line HaCaT (human immortalized keratinocyte cell line).

While complex **38**, [FeCl_2_(Tpm)], did not exhibit any activity against HCT116 and HaCaT cancer cell lines, it promoted the B16 cell line, whose viability increased with its presence. On the other hand, complex **39**, [Co(Tpm^OH^)_2_](NO_3_)_2_, exhibited low cytotoxic effects against the B16, HCT116, and HaCaT cell lines, with IC_50_ values of 88, 500, and 380 μM, respectively. The ability of the compounds to stimulate or suppress cell migration was studied through scratch assays. Unlike cytotoxicity, both complexes exhibited anti-mitogenic effects at non-toxic concentrations. The iron(II) complex was able to decrease the ability of HCT116 and HaCaT cell lines to migrate, but enhanced the migration rate of B16 cells, while cobalt(II) complex **39** inhibited the cell migration ability of all tested cell lines. This anti-motogenic ability of **39** indicates a potential antimetastatic activity.

As with many other types of potential drug leads, these results illustrate that scorpionates, while being developed for anti-tumoral agents, sometimes display the opposite activity. The tested iron complex (**38**) inhibits cell migration in human cell lines (HaCaT and HCT116) but promotes it in the murine (B16) cell line, and displays no cytotoxicity against the tested human cell lines but enhances the viability of the murine cell line. These hard-to-reconcile results suggest that more focused or larger panels of cell lines, as well as expanded sets of activity assays, need to be systematically employed to accurately assess the biological activity of these metal complexes.

An analogue Ru(II) complex with tris(pyrazol-1-yl)methane has also been studied [49]. The cytotoxicity of nine Ru(II) tpm complexes (**40**–**48**, Figure 5) was evaluated against human cervical carcinoma (HeLa), colorectal carcinoma (HCT116), rhabdomyosarcoma (RD), breast cancer (MCF-7), and skin melanoma (518A2) cell lines, as well as against non-tumoral human fibroblasts (MRC5pd30), using cisplatin as a control [55]. All compounds exhibited anticancer activity in the micromolar range, and Ru(II) scorpionates **46** and **47** exhibited antiproliferative activity comparable to that of cisplatin. The highest cytotoxic effect was shown by compound **40,** which was two to three times higher than the one exhibited by cisplatin (Figure 5B). Furthermore, all of the complexes have shown better selectivity toward cancerous cell lines over noncancerous ones compared to cisplatin (Figure 5C).

To complement the cell viability study, the mechanism of inhibiting the growth of cancer cells was also investigated. The primary mechanism of action of this Ru(II) Tpm class of complexes was found to be the disruption of calcium homeostasis, specifically by inhibiting mitochondrial calcium intake. Although ruthenium complexes are known to affect many different metabolic pathways of cells, this is the first study that establishes the direct involvement of mitochondrial calcium homeostasis regulation in the biological activity of Ru complexes against cancer cells.

Ru(II) complexes are particularly used for their anti-tumoral properties. Maintaining the Ru(II)-tpm scaffold, some κ^2^-complexes with bis(diphenylphosphino)alkanes (C2 to C4) (**49**–**54**, Figure 5) display an interesting ability to inhibit the growth of MCF-7 (breast) and HeLa (cervical) cancer cell lines, with IC_50_ values below 10 μM (vs. 12.4 μM and above for cisplatin, Figure 5D) [56].

**Figure 6:**
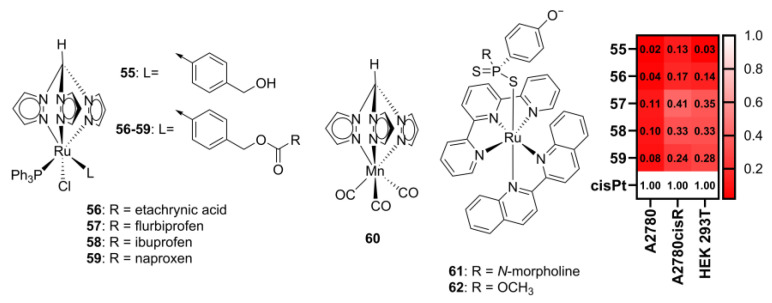
The structure of Ru(II) complexes with ligands with inherent bioactivity and their relative cytotoxicity. The relative cytotoxicity was computed from the results published in the original works as the ratio of IC_50_ for cisplatin divided by IC_50_ for the complex; the higher the value, the higher the activity of the complex (relative to cisplatin, white). Values in red are less active than cisplatin, while values in green are more active than cisplatin. cisPt—cisplatin. The original cytotoxicity data are given in the Supplementary Materials (Table S9).

The functionalized Tpm complexes discussed in this section offer an interesting approach to widen the biological activity of scorpionate complexes by allowing for the inclusion of a covalently bound active moiety, potentially affording a drug with combined mechanisms of action. The inclusion of molecules with established biological activity has also been demonstrated beyond functionalization of the tpm ligand via direct coordination of the metal. For example, Ru-tpm complexes with anti-inflammatories flurbiprofen, ibuprofen, and naproxen, or the glutathione transferase (GST) inhibitor ethacrynic acid (**55**–**59**, Figure 6), have been shown to be water-stable and to display antiproliferative activity against human ovarian carcinoma (A2780), cisplatin-resistant ovarian carcinoma, and embryonic kidney (HEK 293T) cell lines in the 4 to 20 mM range [60]. While not designed primarily for targeting cancer, compounds **55** to **59** are cytotoxic toward cancer cell lines, albeit about 10x less than cisplatin (Figure 6), while also displaying improved COX-2 inhibitory activity.

Another pathway toward multiple-functional drugs involves the use of Ru-tpm complexes that leverage the remaining available Ru coordinating positions to coordinate, for example, carbon monoxide (**60**, Figure 6) [61,62] or H_2_S-donating ligands (**61**–**62**, Figure 6) [63], affording controllable light-triggered gas-releasing systems that can be used for in situ CO and H_2_S release in living systems.

## 5. Poly(pyrazol-1-ylmethyl)amine Complexes

**Figure 7:**
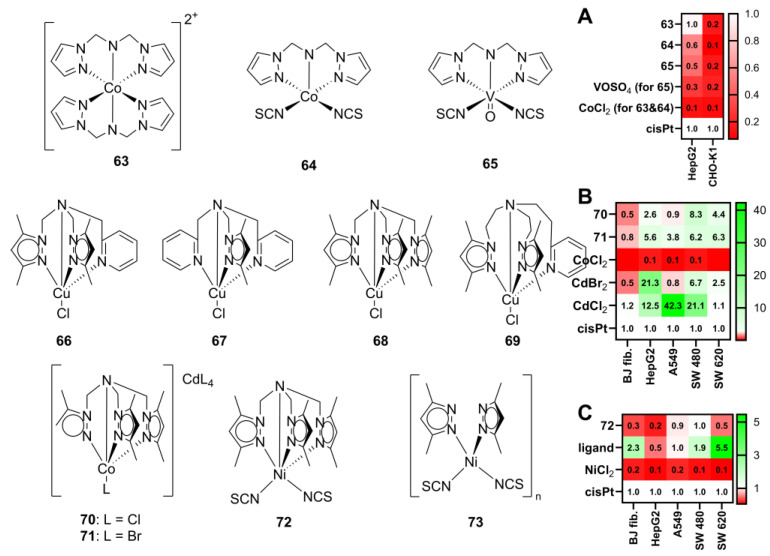
The structure of scorpionate complexes with poly(pyrazol-1-ylmethyl)amines ligands. Adapted from [20,64,65,66]. (**A**) The relative cytotoxicity of compounds **63**–**65**, computed from the values of Tyszka-Czochara et al. [20]. (**B**) The relative cytotoxicity of compounds **70**–**71**, computed from the values of [66]. (**C**) The relative cytotoxicity of **72**, computed from the values of [66]. The relative cytotoxicity was computed from the results published in the original works as the ratio of IC50 for cisplatin divided by IC50 for the complex; the higher the value, the higher the activity of the complex (relative to cisplatin, white). Values in red are less active than cisplatin, while values in green are more active than cisplatin. cisPt—cisplatin. The original cytotoxicity data are given in the Supplementary Materials (Tables S10–S12).

Cobalt and vanadium scorpionate complexes, with a dipolar tridentate *N*,*N-*bis(3,5-dimethylpyrazol-1-ylmethyl)amine ligand (**63**–**65**, Figure 7), have also been studied [20]. The cytotoxic effects of the complexes were tested against the human liver Hep G2 cancer cell line and the non-tumoral Chinese hamster ovary cell line CHO-K1. Complexes **63** and **64** exhibited promising cytotoxicity (Figure 7A) against the Hep G2 cancer cell line, with an IC_50_ value of 22 µM for complex **63** (comparable to that of cisplatin, 21.3 µM) and an IC_50_ value of 38 µM for complex **64**. The vanadium complex **65** displayed lower anticancer activity, with an IC_50_ value of 45.6 µM, nearly twice that of cisplatin. Despite this, all of the complexes exhibited better selectivity toward cancer cells than cisplatin, with antiproliferative indices of 5.5 for **63**, 7.0 for **64**, and 2.7 µM for **65**, while cisplatin had a selectivity index of only 0.9 µM. Flow cytometry studies showed that these complexes induce cell death through different mechanisms. While cobalt complexes **63**–**64** predominantly initiated cancer cell death through necrosis, complex **65** demonstrated the ability to induce apoptosis. Moreover, real-time PCR analysis demonstrated that all of the complexes are able to regulate the expression of vascular endothelial growth factor (VEGF) and matrix metalloproteinases MMP-2 and MMP-9, suggesting their potential in not only killing cancer cells but also in targeting mechanisms that contribute to tumor growth and metastasis.

Copper(II) complexes with similar ligands (**66**–**69**, Figure 7) were also studied for their cytotoxic effects against ovarian cancer (A2780), cisplatin-resistant (A2780R), human osteosarcoma (HOS), and colon carcinoma (CaCo-2) cell lines [64], compared to the standard chemotherapeutics cisplatin, oxaliplatin, and carboplatin.

While oxaliplatin and carboplatin showed no significant cytotoxicity against the tested lines, cisplatin demonstrated moderate activity, particularly against A2780, A2780R, and HOS, with IC_50_ values of 20.1 μM, 45.7 μM, and 47.4 μM, respectively. Complex **69** exhibited the highest activity among all tested compounds, with IC_50_ values as low as 1.4 μM for A2780, and significantly lower than cisplatin for A2780R and HOS, at 8.3 μM and 4.7 μM, respectively.

Further studies were conducted to assess the metabolic stability and protein interactions of complexes **66** and **69**. Complex **66** was shown to degrade in the presence of L-cysteine, losing its 3,5-dimethylpyrazolylmethane ligand and forming a stable adduct with cytochrome c. Complex **68**, on the other hand, did not exhibit any signs of degradation with L-cysteine or glutathione and was able to form two types of adducts with cytochrome c. This difference in stability and protein interaction is likely to reflect on the different cytotoxic activity of the complexes, offering insights into their potential therapeutic mechanisms.

Cobalt complexes with a cadmium counter ion (**70**–**71**, Figure 7) were also analyzed for their anticancer activity [65] against colorectal adenocarcinoma (SW480 and SW620), hepatocellular carcinoma (HepG2), and lung carcinoma (A549) cell lines, comparing their effect on noncancerous fibroblasts (BJ).

The results revealed promising anticancer activity, with both complexes displaying IC_50_ values in the low micromolar range: 8.2 (HepG2), 18.1 (A549), 3.3 (SW480), and 2.7 µM (SW620) for complex **70**, and 3.8 (HepG2), 4.5 (A549), 4.4 (SW480), and 1.9 µM (SW620) for complex **71**. Importantly, the activity of complex **71** surpassed that of cisplatin across most tested cancer cell lines (Figure 7B), also demonstrating superior selectivity. However, both complexes displayed lower cytotoxicity than that of the isolated cadmium salts and were nearly as toxic to noncancerous BJ fibroblasts. This may suggest that the primary source of cytotoxicity might stem from the anionic component of the complex, prompting further developments in the synthesis of these complexes.

Nickel(II) complexes of tris(3,5-dimethylpyrazol-1-ylmethyl)amine and 3,5-dimethylpyrazole ligands (**72** and **73**, Figure 7) were also investigated for their cytotoxic potential against the same cell lines [66].

The preliminary cytotoxicity study with SW610 and BJ cell lines showed that the Ni complex **72** is 10 times more toxic toward colorectal adenocarcinoma and at the same time 8-fold less toxic against the fibroblast cell line compared to the pyrazole complex **73**. Furthermore, a comparison of complex **72** with cisplatin revealed that the Ni complex exhibited a cytotoxicity profile similar to that of cisplatin for SW480 and A549 cells (Figure 7C). However, its activity against SW620 was lower than that of cisplatin, and it showed no significant effect against HepG2. Notably, complex **72** was found to be three times less toxic to fibroblasts compared to cisplatin, with IC_50_ values of 40.8 µM and 13.0 µM, respectively. This suggests that complex **72** possesses higher selectivity toward cancer cells over noncancerous cells, highlighting its potential as a selective anticancer agent.

Flow cytometry analysis of the SW620 cell line provided insights into the cell death mechanisms induced by both Ni complexes. Complex **72**, at 100 µM, predominantly induced apoptosis, with 36.7% of cells in early apoptosis and 61.9% in late apoptosis. Conversely, the Ni pyrazole complex **73** induced necrosis in 30% of cells, with only 18% undergoing apoptosis. In comparison, cisplatin induced apoptosis in 82.1% of cells and necrosis in 15.4%. These findings suggest that complex **72** not only exhibits potent cytotoxicity but predominantly promotes apoptosis (over necrosis), a preferred mechanism for anticancer therapies [67].

## 6. Conclusions

In this review, we sought to highlight the emerging potential of various scorpionate complexes and their ligands, particularly in the context of anticancer research. While these compounds have already demonstrated a wide range of biological activities, their full potential in biomedical applications, especially cancer therapy, remains underexplored.

The simplicity of their scaffolds contrasts with the complexity of their biological activities. Predicting activity from structure often proves challenging, as subtle changes in ligand design can lead to disproportionate shifts in efficacy.

Copper complexes show strong anticancer activities across various studies, indicating that their redox activity can be useful for targeting cancer cells. Zinc complexes also exhibit promising activity, especially against difficult-to-treat cancers like triple-negative breast cancer. Despite being redox-inactive, zinc complexes exhibit DNA/protein interactions that may contribute to their cytotoxicity.

Ruthenium complexes demonstrate selective toxicity by disrupting mitochondrial calcium homeostasis, a unique mechanism that may afford specificity to cancer cells. Silver complexes show significant potency, particularly in resistant cancer cell lines, suggesting that they could be valuable in targeting drug-resistant cancers.

The exact mechanisms of action of these compounds, however, remain mostly speculative, further illustrating the unpredictability of these systems. These findings challenge conventional views of redox-mediated activity and emphasize the unique therapeutic avenues that scorpionates can offer.

Bis(pyrazol-1-yl)borate ligands and derivatives with sulfur-containing heterocycles (e.g., 2-mercaptobenzimidazole) tend to increase the stability and cytotoxicity of copper complexes, indicating that sulfur ligands may enhance coordination stability and bioactivity. Unsubstituted pyrazoles often yield complexes with higher cytotoxic activity than those with bulkier or substituted ligands, likely due to improved cellular uptake and interactions with biomolecules, but ligand selection is still a trial-and-error approach. Dipodal ligands such as tridentate amines confer selective cytotoxicity and, in cobalt and vanadium complexes, modulate gene expression related to metastasis, indicating potential antimetastatic properties. The unpredictable nature of scorpionate activity requires reimagining the possibilities of ligand design, transforming the yet unpredictable and often elusive and serendipitous activity of scorpionates into systematic approaches for future work. In fact, most of the assayed scorpionate complexes display cytotoxic activity which is, at best, comparable to that of cisplatin, and no formal structure–activity relationships have been put forward to guide the synthesis of complexes with superior activity.

Regarding the mechanism of action, for redox-active metals like copper, complexes that facilitate ROS production and/or inhibit thioredoxin reductase (TrxR) can enhance cancer cell cytotoxicity. Designing complexes that optimize these mechanisms may improve anticancer efficacy. Selectivity may come from mechanisms such as the observed mitochondrial disruption in Ru complexes or paraptosis (an alternative to apoptosis), making these complexes valuable tools for cancers with apoptosis resistance. Targeting pathways like mitochondrial calcium regulation or inducing endoreticulum stress may enhance their selectivity toward cancer cells.

These ligands have shown significant success in recent studies; however, further refinement and optimization could greatly enhance their therapeutic efficacy. Continued research focusing on their structural diversity, metal coordination properties, and biological interactions could pave the way for the development of highly potent and selective anticancer agents, positioning scorpionate complexes as promising candidates in next-generation therapeutic strategies.

## Data Availability

No new data were created or analyzed in this study.

## Supplementary Materials

The following supporting information can be downloaded at https://www.mdpi.com/article/10.3390/molecules29235672/s1: The original cytotoxicity data from the cited references, Tables S1–S5: Cytotoxicity data for poly(pyrazol-1-yl)borate complexes (Section 3); Tables S6–S9: Cytotoxicity data for poly(pyrazol-1-yl)methane complexes (Section 4); Tables S10–S12: Cytotoxicity data for poly(pyrazol-1-yl)amine complexes (Section 5).

## Abbreviations

2008	human ovarian carcinoma cell line
518A2	human metastatic melanoma cell line
A431	human cervical carcinoma cell line
A549	human lung adenocarcinoma epithelial cell line
A2780	human ovarian cancer cell line
A2780R	cisplatin-resistant human ovarian cancer cell line
B16	mouse melanoma cell line
BJ	human fibroblast cell line
C13	cisplatin-resistant human ovarian cancer cell line
CaCo-2	human colorectal adenocarcinoma cell line
Caki-2	human renal carcinoma cell line
CHO-K1	Chinese hamster ovary cell line
H460	human lung carcinoma cell line
HaCaT	human immortalized keratinocyte cell line
HCC1937	human breast cancer cell line
HCT116	human colon cancer cell line
HCT-15	human colorectal cancer cell line
HeLa	human cervical cancer cell line
HepG2	human hepatocellular carcinoma cell line
HOS	human osteosarcoma cell line
Hs 578T	human breast cancer cell line
MCF-7	human breast cancer cell line
MDA-MB-231	human breast cancer cell line
MDA-MB-468	human breast cancer cell line
MRC5pd30	Medical Research Council human fibroblast cell line 5
NMDA	*N*-methyl-D-aspartate
PCy3	tricyclohexylphosphine
PPh3	triphenylphosphine
PTA	1,3,5-triaza-7-phosphadamantane
PSN-1	human breast cancer cell line
RD	human rhabdomyosarcoma cell line
ROS	reactive oxygen species
SKBR3	human breast cancer cell line
SW620	human colon cancer cell line
SW480	human colon cancer cell line
SW116	human colorectal cancer cell line
T24	human bladder cancer cell line
TNBC	triple-negative breast cancer
Tp	tris(pyrazol-1-yl)borate
TrxR	thioredoxin reductase
U1285	human breast cancer cell line
PC3	human prostate cancer cell line
