# Supplementary material for: The Elusive Biological Activity of Scorpionates: A Useful Scaffold for Cancer Therapy?
Source: Molecules. 2024 Nov 30;29(23):5672. doi: 10.3390/molecules29235672 (PMC11643361; doi:10.3390/molecules29235672)
Supplement: Supplementary file 1 [file molecules-29-05672-s001.zip › molecules-3327181-supplementary.pdf]

The Elusive Biological Activity of Scorpionates: A Useful Scaffold for Cancer Therapy?

Artem Petrosian, Pedro F. Pinheiro, Ana P. C. Ribeiro, Luísa M. D. R. S. Martins * and Gonçalo C. Justino *

* Correspondence: luisammartins@tecnico.ulisboa.pt (L.M.D.R.S.M.); goncalo.justino@estbarreiro.ips.pt (G.C.J.)

Supplementary Information

Contents

Tables S1 to S12 - Tables with cytotoxicity data from the original data cited in this work 2

References 9

Table S1– Cytotoxicity of compounds **10-15** assessed by the standard MTT assay. Original values from Faghig et al. [1] used for Figure 4A in the main text. SD – standard deviation.

Compound		Cell lines					
		SW1116		A549		MCF-7	
This work	Original work	IC_{50} (μM)	SD	IC_{50} (μM)	SD	IC_{50} (μM)	SD
**10**	1	0.65	0.01	11.44	0.52	1.18	0.12
**11**	2	19.8	2.2	133.5	0.73	75.29	3.07
**12**	3	44.74	1.52	92.68	0.75	104.08	11.29
**13**	4	7.63	1.17	9.98	0.62	1.42	0.06
**14**	5	77.66	2.44	188.68	52.34	121.7	23.42
**15**	6	47.43	2.3	174.58	1.88	220.43	2.21
cisplatin	cisplatin	8.62	0.3	33.52	2.72	61.56	3.1

Table S2– Cytotoxicity of compounds **16-19** assessed by the standard MTT assay on the MCF-7 cell line. Original values from Ghorbanpour et al. [2] used for Figure 4B in the main text. SD – standard deviation.

Complex				Ligand only			
This work	Original work	IC_{50} (μM)	SD	This work	Original work	IC_{50} (μM)	SD
**16**	5	25.37	0.41	-	1	70.87	1.76
**17**	6	44.21	2.44	-	2	85.94	1.5
**18**	7	30.4	1.09	-	3	83.17	0.57
**19**	8	37.2	0.28	-	4	80.51	0.72
cisplatin		100.38	0.52	-			

Table S3 – Cytotoxicity of compounds **20-21** assessed by the standard MTT assay. Original values from Narwane et al. [3] used for Figure 4C in the main text. SD – standard deviation.

Compounds		Cell lines							
		MDA-MB-231		MDA-MB-468		HCC1937		Hs578T	
This work	Original work	IC_{50} (μM)	SD	IC_{50} (μM)	SD	IC_{50} (μM)	SD	IC_{50} (μM)	SD
**20**	1	6.81	0.98	16.56	1.32	13.54	1.77	12.51	1.84
**21**	2	8.85	1.05	10.85	1.72	10.6	1.04	6.68	1.16

Table S4– Cytotoxicity of compounds **23-36** assessed by the standard MTT assay. Original values from Gandin et al. [4] used for Figure 4D in the main text. SD – standard deviation.

			Cell lines														
Compound			MCF-7		A431		HCT-15		A375		BxPC3		SH-SY5Y		A549	
	This work	Original work	IC_{50} (μM)	SD	IC_{50} (μM)	SD	IC_{50} (μM)	SD	IC_{50} (μM)	SD	IC_{50} (μM)	SD	IC_{50} (μM)	SD	IC_{50} (μM)	SD
[HB(pz)$_3$]Cu(PCN) (**1**)	**23**	1	0.73	0.17	0.41	0.18	0.99	0.54	0.65	0.23	0.62	0.46	0.77	0.83	0.84	0.25
[HB(pz)$_3$]Cu(PTA) (**2**)	**24**	2	0.79	0.38	1.88	1.7	2.12	1.13	1.85	1.82	2.24	1.1	1.45	1.71	1.49	1.91
[HB(pz)$_3$]Cu(DAPTA) (**3**)	**25**	3	9.59	2.23	6.65	1.7	11.83	2.26	7.57	2.11	7.64	1.41	8.46	2.28	13.13	1.25
[(HB(pz)$_3$)Cu(PTA-SO$_2$)] (**4**)	**26**	4	2.12	1.06	1	1.16	2.01	1.05	2.25	0.47	1.43	0.97	2.26	0.75	3.65	1.02
[HB(pz)$_3$]Cu(thp) (**5**)	**27**	5	ND		ND		ND		ND		ND		ND		ND	
[HB(pz)$_3$]Cu(PPh$_3$) (**6**)	**28**	6	3.01	0.23	1.98	1.11	2.99	1.54	3.17	1.07	1.45	1.73	3.13	1.96	3.17	0.48
[HB(pz)$_3$]Cu[P(p-C$_6$H$_4$F)$_3$] (**7**)	**29**	7	3.23	1.32	2.34	1.02	0.99	0.36	2.97	0.54	0.76	0.37	1.12	0.42	2.22	1.31
[HB(3-(CF$_3$)pz)$_3$]Cu(PCN) (**8**)	**30**	8	3.61	1.41	7.92	1.06	4.25	1.61	8.14	1.32	7.96	1.87	11.34	1.46	6.54	2.23
[HB(3,5-Me$_2$pz)$_3$]Cu(PCN) (**9**)	**34**	9	8.02	2.36	9.27	1.35	10.15	1.57	5.63	1.25	5.74	2.15	5.36	1.19	10.08	2.54
[HB(3-(NO$_2$)pz)$_3$]Cu(PCN) (**10**)	**31**	10	13.23	2.52	5.43	1.75	11.24	1.64	8.21	1.79	6.47	2.38	7.43	1.65	8.19	2.11
[HB(Btz)$_3$]Cu(PCN) (**11**)	**36**	11	19.25	1.96	11.12	2.34	12.56	2.45	9.64	238	16.27	4.09	14.72	3.25	15.56	2.28
[HB(3-(CF$_3$)pz)$_3$]Cu(PTA) (**12**)	**32**	12	4.45	1.02	15.02	1.98	6.48	2.98	8.47	2.22	7.46	2.17	11.14	2.13	7.77	2.12
[HB(3,5-Me$_2$pz)$_3$]Cu(PTA) (**13**)	**35**	13	6.98	1.52	9.83	2.09	8.64	1.74	7.02	1.51	9.53	2.35	12.66	1.99	8.32	1.22
[HB(3-(NO$_2$)pz)$_3$]Cu(PTA) (**14**)	**33**	14	17.33	2.36	8.28	2.24	23.78	2.11	17.45	3.11	7.53	2.81	8.41	3.07	22.51	2.25
[HB(Btz)$_3$]Cu(PTA) (**15**)	**34**	15	11.12	2.65	10.25	3.14	4.65	1.76	12.72	2.96	8.36	2.11	14.27	3.93	19.76	1.71
PPh$_3$			34.18	3.35	47.53	1.14	54.13	4.25	52.31	2.46	62.24	2.17	41.24	2.94	30.12	2.13
PTA			100	100	100	100	100	100	100	100	100	100	100	100	100	
PCN			100	100	100	100	100	100	100	100	100	100	100	100	100	
PTA-SO$_2$			69.46	3.35	61.12	4.36	100	100	100	100	100	100	100	100	100	
DAPTA			85.32	3.95	100		67.12	3.14	100		89.53	4.13	100		100	

$[P(p\text{-}C_6H_4F)_3]$		63.32	3.99	56.04	3.46	100		86.11	4.86	100		100		56.45	4.42
$Na[HB(pz)_3]$		100		40.44	1.44	77.58	1.97	66.87	2.34	75.37	3.74	97.43	2.16	100	
$Na[HB(3\text{-}(CF_3)pz)_3]$		67.39	2.73	46.47	3.27	58.72	2.25	100		100		100		100	
$K[HB(3,5\text{-}Me_2pz)_3]$		63.32	2.39	42.26	1.98	58.79	2.19	68.27	3.84	73.35	5.01	98.48	3.33	100	
$Na[HB(Btz)_3]$		100		100		100		100		100		100		100	
$Na[HB(3\text{-}(NO_2)pz)_3]$		49.31	3.24	66.09	2.98	100		69.63	2.12	44.48	2.84	59.16	4.19	100	
cisplatin	cisPt	7.6	0.21	1.65	0.51	16.65	2.63	3.11	0.98	10.17	1.65	5.36	1.74	12.64	0.81

Table S5– Cytotoxicity of compounds **23-36** assessed by the standard MTT assay. Original values from Gandin et al. [4] used for Figures 4E and 4F in the main text. SD – standard deviation.

Compounds			Cell lines									
			2008		C13*		.	LoVo		LoVo	MDR	
	This work	Original work	IC$_{50}$ (μM)	SD	IC$_{50}$ (μM)	SD	R.F	IC$_{50}$ (μM)	SD	IC$_{50}$ (μM)	SD	R.F.
[HB(pz)$_3$]Cu(PCN) (**1**)	**23**	1	0.29	0.09	0.39	0.11	1.3	1.41	0.56	0.84	0.31	1.3
[HB(pz)$_3$]Cu(PTA) (**2**)	**24**	2	1.05	0.76	1.45	0.6	1.4	2.46	1.01	2.11	0.93	1.4
[HB(pz)$_3$]Cu(DAPTA) (**3**)	**25**	3	9.11	1.15	8.86	1.3	1	7.35	2.14	8.65	1.25	1.2
[HB(pz)$_3$]Cu(PTA-SO$_2$) (**4**)	**26**	4	2.65	1.36	1.95	1.63	0.7	2.25	0.74	1.62	1.03	0.7
[HB(pz)$_3$]Cu(PPh$_3$) (**6**)	**28**	6	3.21	2.11	3.92	1.22	1.2	3.28	0.85	2.41	1.15	0.6
[HB(pz)$_3$]Cu[P(p-C$_6$H$_4$F)$_3$] (**7**)	**29**	7	2.16	0.98	2.25	0.95	1	3.54	1.11	2.65	0.87	0.7
[HB(3-(CF$_3$)pz)$_3$]Cu(PCN) (**8**)	**30**	8	2.11	0.96	2.21	0.62	1	6.14	1.73	6.03	1.33	1
[HB(3,5-Me$_2$pz)$_3$]Cu(PCN) (**9**)	**34**	9	3.37	0.97	3.23	1.01	0.9	4.88	1.98	5.05	1.12	1
[HB(3-(NO$_2$)pz)$_3$]Cu(PCN) (**10**)	**31**	10	7.36	3.14	8.46	2.26	1.1	11.26	3.16	12.53	2.98	1.1
[HB(Btz)$_3$]Cu(PCN) (**11**)	**36**	11	8.65	2.23	7.84	1.32	0.9	11.73	2.43	12.63	1.64	1.1
[HB(3-(CF$_3$)pz)$_3$]Cu(PTA) (**12**)	**32**	12	3.26	1.05	2.89	0.92	0.9	5.46	1.31	5.44	1.65	0.9
[HB(3,5-Me$_2$pz)$_3$]Cu(PTA) (**13**)	**35**	13	3.04	1.23	3.52	1.26	1.1	5.32	1.54	4.43	1.75	0.8
[HB(3-(NO$_2$)pz)$_3$]Cu(PTA) (**14**)	**33**	14	5.47	2.13	6.88	0.92	1.3	7.58	3.14	8.85	2.95	1.1
[HB(Btz)$_3$]Cu(PTA) (**15**)	**34**	15	3.54	1.51	3.77	0.94	1.1	4.43	0.93	5.15	1.09	1.2
cisplatin			2.22	1.03	22.77	2.01	10.2					
doxorubicin								1.11	0.86	19.21	2.37	17.3

Table S6– Cytotoxicity of compounds **23-36** assessed by the standard MTT assay. Original values from Morelli et al. [5] used for Figure 5A in the main text. SD – standard deviation.

Compounds		Cell lines											
		MCF7		SKBR3		H460		T24		PC3		Caki-2	
This work	Original work	IC_{50} (μM)	SD	IC_{50} (μM)	SD	IC_{50} (μM)	SD	IC_{50} (μM)	SD	IC_{50} (μM)	SD	IC_{50} (μM)	SD
NMDAR antagonist	1	70	3.5	32	1.8	34	2.9	170	5.3	52	2.8	145	5.8
23	2	ND	ND	ND	ND								
24	3	ND	ND	ND	ND								
Ligand for 25	4	148	5.2	160	4.3	144	6.1	> 300		>300			
Ligand for 26	5	49	2.8	50	2.1	31	2.2	34	2.1	53	2.3	137	4.9
25	6	107	4.6	86	3.7	60	3.6	110	4.5	85	4.7	75	3.4
26	7	25	1.3	32	1.9	15	1.1	29	2	21	1.4	18	1.7
	cisplatin	8.3	1.2	16	1.5	1.6	0.8	0.7	0.1	10	1.9	1.4	0.7

Table S7– Cytotoxicity of compounds **40-48** assessed by the standard MTT assay. Original values from Cervinka et al. [6] used for Figures 5B and 5C in the main text. SD – standard deviation; SI – selectivity index.

Compounds		Cell lines												SI
		MCF-7		HeLa		518A2		HCT116		RD		MRC5pd30		
This work	Original work	IC50 (mM)	SD	IC50 (mM)	SD	IC50 (mM)	SD	IC50 (mM)	SD	IC50 (mM)	SD	IC50 (mM)	SD	
**40**	1	2.4	0.6	4.0	0.4	2.6	0.4	1.5	0.1	2.2	0.2	5.8	0.7	2.3
**41**	2	32	4	53	4	26	4	25	2	26	5	76	1	2.4
**42**	3	38	8	91	3	33	5	25	1	27	4	83.6	0.5	2
**43**	4	37	7	36	1	35	4	25	2	25	3	56.6	0.7	1.8
**44**	5	32	5	54	6	31	3	30	2	23	2	63	4	1.9
**45**	6	46	7	54	14	38	8	31	2	38	6	82	4	2
**46**	7	6	1	10	2	6.8	0.8	6.7	0.4	6	1	24	1	3.4
**47**	8	10	2	15	1	10	2	8	2	6.6	0.7	19.7	0.4	2
**48**	9	43	7	36	2	38	7	35	6	24	3	50.8	0.4	1.5
	cisplatin	13	3c	14	3c	2.6	0.7d	8	1c	4.6	0.3	11.7	0.8c	1.1

Table S8– Cytotoxicity of compounds **49-54** assessed by the standard MTT assay. Original values from Walker et al. [7] used for Figure 5D in the main text. SD – standard deviation.

Compounds		Cell lines			
		MCF7		HeLa	
This work	Original work	IC50 (mM)	SD	IC50 (mM)	SD
**49**	1	>50		>50	
**50**	2	>50		>50	
**51**	3	8.1	4.6	4	0
**52**	4	2.9	0.07	6.9	1.31
**53**	5	2.9	0.07	5.8	0.35
**54**	6	4.7	0.07	7.4	0.21
	Control	0.8	0	1.4	0.07
	cisplatin	18		12.4	0.85

Table S9– Cytotoxicity of compounds **55**-**59** assessed by the standard MTT assay. Original values from Gobbo et al. [8] used for Figure 6A in the main text. SD – standard deviation.

Compounds		Cell line					
		A2780		A2780cisR		HEK 293T	
This work	Original work	IC_{50} (μM)	SD	IC_{50} (μM)	SD	IC_{50} (μM)	SD
	Ligand						
-	2	8	3	15	9	10	4
**55**	3	26	10	26	5	47	8
**56**	4	12	2	20	5	10	3
**57**	5	4.5	0.6	8	2	4	2
**58**	6	5	1	10	2	4.3	0.8
**59**	7	6	1	14	4	5	1
	EA	38	2	38	10	27	11
	FLU						
	IBU					85	6
	NAP					100	2
	cisPt	0.5	0.1	3.3	0.5	1.4	0.9
	RAPTA-C	99	3				

Table S10– Cytotoxicity of compounds **63**-**65** assessed by the standard MTT assay. Original values from Tyszka-Czochara et al. [9] used for Figure 7A in the main text. SD – standard deviation.

Compounds		Cell lines			
		HepG2		CHO-K1	
This work	Original work	IC_{50} (μM)	SD	IC_{50} (μM)	SD
**63**	1	22	4	121.8	10
**64**	2	38.2	5	268.2	15
**65**	3	45.6	5	123.1	3
metal for 43	VOSO4	75.6	8	98.3	9
metal for 41, 42	COCL2	160.2	18	175.6	28
cisplatin		21.3	2	19.1	2

Table S11– Cytotoxicity of compounds **70-71** assessed by the standard MTT assay. Original values from Adach et al. [10] used for Figure 7B in the main text. SD – standard deviation.

Compounds		Cell lines									
		BJ fibroblasts		HepG2		A549		SW 480		SW 620	
This work	Original work	IC_{50} (μM)	SD	IC_{50} (μM)	SD	IC_{50} (μM)	SD	IC_{50} (μM)	SD	IC_{50} (μM)	SD
**70**	1	24.2	2.9	8.2	0.5	18.1	2	3.3	0.3	2.7	0.3
**71**	2	17.1	2.1	3.8	0.5	4.5	0.3	4.4	0.3	1.9	0.4
	CoCl2	282.5	28.4	160.2	18.1	281.5	16.7	228.1	8.7	256.4	23.9
	CdBR2	25.9	3	1	0.1	20.1	4.8	4.1	0.1	4.8	0.5
	CdCl2	11.1	0.9	1.7	0.1	0.4	0.1	1.3	0.2	10.5	1.6
cisplatin		13	1.9	21.3	2.4	16.9	1.2	27.4	2.8	12	1.1

Table S12– Cytotoxicity of compound **72** assessed by the standard MTT assay. Original values from Adach et al. [11] used for Figure 7C in the main text. SD – standard deviation.

Compounds		Cell lines									
		BJ fibroblasts		HepG2		A549		SW 480		SW 620	
This work	Original work	IC_{50} (μM)	SD	IC_{50} (μM)	SD	IC_{50} (μM)	SD	IC_{50} (μM)	SD	IC_{50} (μM)	SD
**72**	1	40.8	4.7	138.4	10.5	18.4	1.3	26.3	3.4	24.8	2.9
	HL1	5.7	0.5	43.1	1.3	17.6	1.1	14.6	1.8	2.2	0.8
	NiCl2	83.7	10.7	306.3	7.4	88.8	8.1	256	28	228.1	29.4
	cisplatin	13	1.9	21.3	2.4	16.9	1.2	27.4	2.8	12	1.1

References

1. Faghih, Z.; Neshat, A.; Mastrorilli, P.; Gallo, V.; Faghih, Z.; Gilanchi, S. Cu(II), Ni(II) and Co(II) complexes with homoscorpionate Bis(2-Mercaptobenzimidazolyl) and Bis(2-Mercaptobenzothiazolyl)borate ligands: Synthesis and in vitro cytotoxicity studies. *Inorganica Chimica Acta* **2020**, *512*, 119896, doi:https://doi.org/10.1016/j.ica.2020.119896.
2. Ghorbanpour, M.; Soltani, B.; Molavi, O.; Shayanfar, A.; Mehdizadeh Aghdam, E.; Ziegler, C.J. Copper (II) complexes based bis(pyrazolyl)borate derivatives as efficient anticancer agents: synthesis, characterization, X-ray structure, cytotoxicity, molecular docking and QSAR studies. *Chemical Papers* **2022**, *76*, 7343-7356, doi:10.1007/s11696-022-02288-9.
3. Narwane, M.; Dorairaj, D.P.; Chang, Y.L.; Karvembu, R.; Huang, Y.H.; Chang, H.W.; Hsu, S.C.N. Tris-(2-pyridyl)-pyrazolyl Borate Zinc(II) Complexes: Synthesis, DNA/Protein Binding and In Vitro Cytotoxicity Studies. *Molecules* **2021**, *26*, doi:10.3390/molecules26237341.
4. Gandin, V.; Tisato, F.; Dolmella, A.; Pellei, M.; Santini, C.; Giorgetti, M.; Marzano, C.; Porchia, M. In Vitro and in Vivo Anticancer Activity of Copper(I) Complexes with Homoscorpionate Tridentate Tris(pyrazolyl)borate and Auxiliary Monodentate Phosphine Ligands. *Journal of Medicinal Chemistry* **2014**, *57*, 4745-4760, doi:10.1021/jm500279x.
5. Morelli, M.B.; Amantini, C.; Santoni, G.; Pellei, M.; Santini, C.; Cimarelli, C.; Marcantoni, E.; Petrini, M.; Del Bello, F.; Giorgioni, G., et al. Novel antitumor copper(ii) complexes designed to act through synergistic mechanisms of action, due to the presence of an NMDA receptor ligand and copper in the same chemical entity. *New Journal of Chemistry* **2018**, *42*, 11878-11887, doi:10.1039/C8NJ01763H.
6. Cervinka, J.; Gobbo, A.; Biancalana, L.; Markova, L.; Novohradsky, V.; Guelfi, M.; Zacchini, S.; Kasparkova, J.; Brabec, V.; Marchetti, F. Ruthenium(II)-Tris-pyrazolylmethane Complexes Inhibit Cancer Cell Growth by Disrupting Mitochondrial Calcium Homeostasis. *J Med Chem* **2022**, *65*, 10567-10587, doi:10.1021/acs.jmedchem.2c00722.
7. Walker, J.M.; McEwan, A.; Pycko, R.; Tassotto, M.L.; Gottardo, C.; Th'ng, J.; Wang, R.; Spivak, G.J. [Tris(pyrazolyl)methane]ruthenium Complexes Capable of Inhibiting Cancer Cell Growth. *European Journal of Inorganic Chemistry* **2009**, *2009*, 4629-4633, doi:https://doi.org/10.1002/ejic.200900766.
8. Gobbo, A.; Pereira, S.A.P.; Biancalana, L.; Zacchini, S.; Saraiva, M.L.M.F.S.; Dyson, P.J.; Marchetti, F. Anticancer ruthenium(ii) tris(pyrazolyl)methane complexes with bioactive co-ligands. *Dalton Transactions* **2022**, *51*, 17050-17063, doi:10.1039/D2DT03009H.
9. Tyszka-Czochara, M.; Adach, A.; Grabowski, T.; Konieczny, P.; Pasko, P.; Ortyl, J.; Świergosz, T.; Majka, M. Selective Cytotoxicity of Complexes with N,N,N-Donor Dipodal Ligand in Tumor Cells. *Int J Mol Sci* **2021**, *22*, doi:10.3390/ijms22041802.
10. Adach, A.; Daszkiewicz, M.; Tyszka-Czochara, M. Comparative X-ray, vibrational, theoretical and biological studies of new in situ formed [CoLSX]2[CdX4] halogenocadmate(II) complexes containing N-scorpionate ligand. *Polyhedron* **2020**, *175*, 114229, doi:https://doi.org/10.1016/j.poly.2019.114229.
11. Adach, A.; Tyszka-Czochara, M.; Bukowska-Strakova, K.; Rejnhardt, P.; Daszkiewicz, M. In situ synthesis, crystal structure, selective anticancer and proapoptotic activity of complexes isolated from the system containing zerovalent nickel and pyrazole derivatives. *Polyhedron* **2022**, *223*, 115943, doi:https://doi.org/10.1016/j.poly.2022.115943.
